# Salt Tolerance in Soybean (*Glycine max* L.): A Comprehensive Review of Molecular Mechanisms, Key Regulators, and Future Perspectives for Saline Soil Utilization

**DOI:** 10.3390/plants14233668

**Published:** 2025-12-02

**Authors:** Tingjia Dong, Lei Yan, Jiahui Wang, Yusheng Niu, Lu Wang

**Affiliations:** 1 Institute of Biomedical Engineering, College of Life Sciences, Qingdao University, Qingdao 266071, China; 2 Research Institute of Modern Agricultural Industry Innovation in Yellow River Delta Saline-Alkali Land, Dongying Vocational College, Dongying 257000, China

**Keywords:** soybean, salt tolerance, saline stress, ion homeostasis, osmotic adjustment, transcription factors, ROS, ABA signaling

## Abstract

Soil salinization poses a significant threat to global agricultural productivity. Among crops, soybean (*Glycine max*), an important source of oil and protein, is more susceptible to salt stress compared to other major crops such as wheat (*Triticum aestivum*) and rice (*Oryza sativa*). To better utilize saline land resources, understanding the mechanisms underlying salt tolerance in soybean is essential for developing new salt-tolerant soybean varieties that contribute to food security. This review synthesizes current knowledge on the molecular mechanisms of salt tolerance in soybean, with a focus on ion homeostasis, osmotic adjustment, oxidative balance restoration, structural adaptations, and transcriptional regulatory networks. Key findings highlight the critical roles of ion transporters—such as GmNHX1, GmSOS1, GmHKT1, and GmCLC1—in maintaining Na^+^/K^+^ and Cl^−^ balance; the accumulation of osmoprotectants like proline and LEA proteins to alleviate osmotic stress; and the activation of antioxidant systems—including SOD, CAT, and APX—to scavenge reactive oxygen species (ROS). Additionally, structural adaptations, such as salt gland-like features observed in wild soybean (*Glycine soja*), and transcriptional regulation via ABA-dependent and independent pathways (e.g., GmDREB, GmbZIP132, GmNAC) further enhance tolerance. Despite these advances, critical gaps remain regarding Cl^−^ transport mechanisms, rhizosphere microbial interactions, and the genetic basis of natural variation in salt tolerance. Future research should integrate genomic tools, omics-based breeding, genome editing techniques such as CRISPR-Cas9, microbial technologies, and traditional breeding methods to develop salt-tolerant soybean varieties, providing sustainable solutions for the utilization of saline–alkali soils and enhancing global food security.

## 1. Background

Soil salinization has become a critical issue and a major constraint on sustainable agricultural development. High evaporation and low precipitation, coupled with unsustainable agricultural practices such as excessive irrigation, poor drainage, and over-fertilization, have led to severe soil salinity accumulation [1]. Approximately 1.4 billion hectares of land worldwide are affected by salinization, with over 1 billion hectares classified as high-risk areas. About 20% of irrigated farmland is impacted by salinity, primarily in regions such as China, Pakistan, Argentina, Central Asia, and West Asia. Notably, China accounts for 10.1% of the world’s saline–alkali land area [2], primarily distributed across arid and semi-arid regions in the central and western areas, the Northeast region, and eastern coastal areas [3]. In Shandong Province, saline–alkali soils are predominantly found in the Yellow River Delta, where the river deposits silt into Bohai Bay, gradually filling the bay and creating new saline–alkali land [4]. For plants, exposure to salt stress triggers a cascade of detrimental physiological effects, including osmotic stress, ionic toxicity, and oxidative damage. These interconnected stresses collectively hinder plant growth, disrupt key developmental processes, and severely reduce crop yields.

Soybean (*Glycine max* (L.) Merr.) is a globally important oilseed crop, providing a primary source of vegetable oil and high-quality plant protein for human consumption and livestock feed [5]. However, China faces two major challenges in soybean production: first, domestic soybean yields remain relatively low, leading to a heavy dependence on imports [6]; second, China has limited per capita arable land, most of which is dedicated to growing staple crops such as wheat (*Triticum aestivum*), rice (*Oryza sativa*), and corn (*Zea mays*) [7]. As a result, conducting in-depth research into the molecular mechanisms underlying soybean salt tolerance, along with the identification, development, and utilization of salt-tolerant genetic resources, holds significant theoretical and practical value. Advancements in these areas will directly contribute to accelerating the breeding of salt-tolerant soybean varieties, promoting the sustainable use of saline soil, and strengthening global food security.

## 2. The Detrimental Impacts of Soil Salinization on Plants and Soybeans

Salt accumulation is a widespread environmental challenge that significantly impacts crop productivity on a global scale [8]. The detrimental effects of salinity on plants can be observed at various physiological and biochemical levels, leading to impaired growth and substantial yield reductions.

### 2.1. Growth Inhibition

At the growth level, high-salt environments directly hinder plant development, leading to a reduction in plant height [9]. Specifically, salt stress impedes cell elongation and disrupts the activity of transport proteins, such as H^+^-ATPase and H^+^-PPase, thereby hindering plant growth [10]. Additionally, elevated salt levels limit nitrogen uptake by plants due to interactions between Cl^−^ and NO_3_^−^, as well as Na^+^ and NH_4_^+^, further affecting plant growth and crop yields [11]. Salt stress impairs plant development at multiple stages: primary root growth declines sharply after salt exposure, entering a phase of stagnation before partially recovering. The stagnation of lateral roots can last for more than two days, with their growth rate decline being significantly more pronounced than that of primary roots [12].

Certain soybean varieties show notable differences in salt sensitivity at different developmental stages. For instance, William 82 seedlings showed growth inhibition under 100–200 mmol/L NaCl treatment but recovered once the stress was removed. A concentration of 300 mmol/L NaCl induced severe growth inhibition without plant mortality. After 6 days of exposure to 400 mmol/L NaCl, the leaves wilted and curled, and recovery was impossible after 11 days of treatment [13]. In contrast, the germination stage exhibits greater tolerance, with a 40% germination rate maintained even when Na^+^ concentrations in the hypocotyl reach 9.3 mg/g fresh weight (FW) [14]. However, there is no inherent correlation between the salt tolerance of seedlings and that of mature plants across different varieties. For example, salt stress during the seedling stage had minimal effect on ‘Lee’, with a significantly smaller reduction in aboveground dry matter during the mature stage compared to other varieties such as ‘Clark 63’ [15].

### 2.2. Photosynthesis and Metabolism

Photosynthesis, the core process of carbon fixation, is significantly disrupted by salt stress. Exposure to high salinity induces stomata closure, reducing intercellular CO_2_ concentration and subsequently limiting the carbon supply essential for photosynthesis. Moreover, salt stress damages the structure of chloroplast and diminishes the levels of photosynthetic pigments, leading to decreased quantum yield and impaired electron transport efficiency within photosystem II (PSII), ultimately lowering the overall photosynthetic rate [16,17]. For example, salt stress severely inhibits strawberry (*Fragaria × ananassa*) growth, partially due to a decline in chlorophyll content, which directly reduces photosynthetic activity [18]. This reduction also affects chlorophyll synthesis and metabolism. High-salinity conditions inhibit the activity of chlorophyll synthetic enzymes, such as porphyrin IX oxidase and magnesium chelating enzyme, while promoting the production of reactive oxygen species (ROS), including superoxide free anions (O_2_^−^) and H_2_O_2_ [19]. These ROS damage thylakoid membranes and chloroplast components, accelerating chlorophyll degradation [20]. For example, salt stress causes a decrease in chlorophyll content in sorghum with a concomitant loss in photosynthetic efficiency [21]. Additionally, salt stress specifically impacts metabolism and crop quality. It notably reduces seed protein content, while its effect on oil content varies depending on the specific variety and environmental conditions [22].

In soybeans, the normal functions of the photosynthetic system were enhanced under 50 mM NaCl stress but inhibited under 150 mM NaCl stress. Under the higher salt concentration, biomass, net leaf photosynthetic rate, stomatal conductance, intercellular carbon dioxide concentration, transpiration rate, chlorophyll fluorescence parameters all decreased [23].

### 2.3. Ion Toxicity and Water Deprivation

Ion homeostasis and water balance are further disrupted under saline–alkali conditions. Excessive Na^+^ and Cl^−^ in the soil hinder the uptake of essential cations such as K^+^ and Ca^2+^, resulting in intracellular ionic imbalance and impairing enzyme activity as well as cellular function [24,25]. In rice, salt stress leads to a marked increase in leaf Na^+^ content and a significant decline in the K^+^/Na^+^ ratio [26]. Additionally, elevated concentrations of soluble salts in the soil lower the water potential of the soil solution, reducing water availability to plants. This induces physiological drought and interferes with normal cellular metabolism [27,28,29].

In soybeans, salt sensitivity is primarily attributed to Na^+^ toxicity rather than Cl^−^. Specifically, reduced seed dry mass to 30% of the control, which is a greater reduction compared to Cl^−^ salts, which only reduced it to 60% of the control. Additionally, NaCl and Na^+^ salts (without Cl^−^) had a more pronounced negative effect on soybean photosynthesis (Pn), reducing it to 50% of the control, compared to Cl^−^ salts (without Na^+^) [30]. The degree of leaf yellowing is positively correlated with Cl^−^ content. Salt-sensitive cultivars tend to accumulate higher Cl^−^ levels than salt-tolerant ones [31,32]. For example, the salt-tolerant cultivar ‘Lee’ maintains lower Na^+^ and Cl^−^ concentrations in its leaves under saline conditions compared with the sensitive cultivars ‘Clark 63’ [15].

### 2.4. Oxidative Damage

Salt stress induces the production of large quantities of ROS, including O_2_^−^, H_2_O_2_, and hydroxyl radical (OH^−^ [33]), which cause significant oxidative damage to plants. These molecules have strong oxidizing properties, leading to DNA mutations, membrane disruption, and the degradation of lipids, proteins, photosynthetic pigments, and other cellular components [34]. For example, in canola (*Brassica napus* L.) under salt stress, accumulation of ROS like H_2_O_2_ results in increased oxidative damage in cells [35]. The oxidative stress resulting from salt stress triggers lipid peroxidation, producing malondialdehyde (MDA), which serves as a biomarker for the extent of lipid peroxidation and reflects the degree of cellular damage [36]. Salt-tolerant varieties can mitigate lipid peroxidation by enhancing antioxidant enzyme activities, thereby enhancing their resilience to salt stress. For instance, under salt stress, the salt-sensitive cultivar (XY15) of rapeseed (*Brassica napus* L.) exhibited significantly greater increases in MDA content in both roots and leaves compared to the salt-tolerant cultivar (HY9). Concurrently, XY15 also displayed higher levels of H_2_O_2_ accumulation and relative electrolyte leakage, indicating more severe lipid peroxidation and membrane damage [37].

In soybean, salt stress suppresses the activity of antioxidant enzymes, disrupts the ROS scavenging equilibrium, and consequently leads to elevated levels of oxidative stress markers such as MDA [5]. Specifically, upon 150 mmol/L NaCl stress in the salt-sensitive soybean cultivar “411”, there is an increase in MDA levels and a decrease in the activities of glutathione reductase (GR), ascorbate peroxidase (APX), catalase (CAT), and superoxide dismutase (SOD) [38]. Another study demonstrated that under mild salinity stress, elevated levels of antioxidant enzymes and a decline in glutathione content can protect nodules against ROS, and hence avoid the breakdown of leghemoglobin and peroxidation of lipid and protein. However, severe salt treatment leads to an increase in GR activity and higher levels of glutathione in its reduced form [39].

### 2.5. Specific Damage to the Symbiotic Nitrogen Fixation System in Soybean

Under salt stress conditions, oxidative damage to soybean root nodules is closely associated with their nitrogen-fixing capacity [38]. Although most research has concentrated on ion and osmotic regulatory mechanisms, the effects of salt stress on plant symbiotic relationships remain underexplored. Nonetheless, it is well-documented that the symbiotic nitrogen fixation (SNF) system between soybeans and rhizobia is highly sensitive to salt stress [40,41]. Salt stress reduces both the number and biomass of nodules and significantly decreases nitrogen-fixing efficiency. Several key mechanisms contribute to this salt-induced disruption of the soybean–rhizobia symbiosis. These include inhibition of rhizobial aerobic respiration, a reduction in leghemoglobin content within nodules, depletion of the energy needed for nitrogen fixation, impeded perception of nodulation factors by root hairs, and inhibition of symbiosis initiation [41,42].

## 3. Molecular Mechanisms of Salt Tolerance in Plants

### 3.1. Salt Signaling in Plants

The plant response to salt stress begins with the perception and early signaling of Na^+^. Non-selective cation channels (NSCCs) are the primary pathway for Na^+^ entry into root cells [43,44], and their activity is regulated by Ca^2+^, cyclic guanosine monophosphate (cGMP), and ROS. Membrane GIPCs synthesized via MOCA1, a glucuronosyltransferase, bind monovalent cations such as Na^+^, subsequently activating Ca^2+^ channels and triggering downstream signals like calcium waves [12]. Early signaling molecules such as Ca^2+^, ROS, and cGMP play crucial roles in initiating and amplifying salt stress signals [45,46,47].

As noted in the review by van Zelm et al., salt stress rapidly increases intracellular Ca^2+^ concentrations, leading to the formation of calcium peaks and waves that can be categorized into fast and late responses. This signaling process also involves the release of Ca^2+^ from vesicles through the TPC1 channel, enabling long-distance signaling. Calcium signaling is decoded by the CBL-CIPK protein complex, with the SOS pathway (SOS3/CBL4-SOS2/CIPK24-SOS1/NHX7) acting as a central component for promoting Na^+^ efflux and maintaining ionic homeostasis [12]. Concurrently, salt stress induces the rapid production of extracellular ROS (e.g., H_2_O_2_), mediated by the RBOH family of NADPH oxidases. ROS play a crucial role in facilitating the intercellular spread of Ca^2+^ signals during salt stress [48]. A positive feedback loop is established between ROS and Ca^2+^: Ca^2+^ activates RBOH to generate ROS, while ROS enhance Ca^2+^ endocytosis, thereby regulating ionic homeostasis and cellular redox balance [48]. Moreover, the early elevation of cGMP enhances Ca^2+^ influx, reduces Na^+^ uptake and K^+^ efflux, effectively enhancing the overall signaling response in coordination with Ca^2+^ and ROS [49]. Together, these tightly integrated signaling pathways enable plants to rapidly adapt to saline conditions by maintaining ionic homeostasis and mitigating oxidative stress.

In addition to ion sensing, changes in the cell wall can be detected by the receptor kinase FERONIA (FER), which plays a role in later signaling events and may contribute to growth regulation during the later stages of the salt stress response [50]. As summarized in the review by to van Zelm et al., under salt stress, the replacement of Ca^2+^ by Na^+^ disrupts pectin cross-linking and leads to cell wall relaxation; the microtubule network is first depolymerized and then reorganized, which affects the localization of cellulose synthesis complexes (CSCs) through the regulation of the PROPYZAMIDE HYPERSENSITIVE1 (PHS1) kinase and the SPIRAL1 (SPR1) protein, and then regulates cell expansion [12]. The receptor kinase FER senses cell wall changes and triggers a late calcium wave that prevents root tip cell swelling and rupture [50].

### 3.2. Hormones Coordinate Growth Stages and Signaling Pathways upon Salt Stress

Salt stress leads to significant changes in plant morphology through dynamic, multistage growth responses and tissue-specific regulation, all modulated by hormonal signals. As described in the review by van Zelm et al., hormones play a vital role in regulating salt tolerance by coordinating ion transport, growth and stress responses, with abscisic acid (ABA) playing a central role [12]. Under salt stress, ABA synthesis is induced in vascular tissues of roots and leaves, primarily through the activation of the *NCED3* gene. The synthesized ABA is transported to guard cells via ABCG25/40 transporters, where it triggers adaptive responses [51,52]. A key response involves the activation of SnRK2.6/OST1, which stimulates the opening of anion channels in guard cells by phosphorylating KAT1 and SLAC1, leading to stomatal closure and reduced water loss [53,54,55]. In addition, under salt stress, ABA signaling initially activates SnRK2 protein kinases to inhibit lateral root growth after emergence from the primary root. However, as the stress continues, the roots recover, and these lateral roots resume growth. Interestingly, ABA has been found to promote the recovery of lateral root growth following this inhibition. This process is mediated by the receptor PYL8, which interacts with transcription factors MYB77, MYB44, and MYB73 to enhance auxin signaling pathway and support lateral root development [56].

In addition to regulating growth and water conservation, ABA activates the expression of genes responsible for synthesizing and accumulating compatible solutes. These substances lower cellular osmotic potential, enabling cells to absorb water from the external environment. Furthermore, they protect enzymes and membrane structures from destabilization caused by high ionic concentrations [57]. Additionally, ABA enhances salt tolerance by upregulating the expression of vacuolar membrane ion transporters, such as *AtNHX1*, and modulating the activity of the Na^+^/H^+^ antiporter via the ABI1 signaling pathway [58,59].

Under salt stress, brassinosteroids (BRs) are perceived at the cell surface primarily by the receptor BRASSINOSTEROID INSENSITIVE 1 (BRI1) [60] and its homologs BRL1 and BRL3 [61]. Upon BR perception by the BRI1-BAK1 receptor complex at the plasma membrane, downstream cytoplasmic kinases, BR-SIGNALING KINASES (BSKs), are activated [62]. This activation triggers the phosphatase BSU1 [63], leading to a defined phosphorylation/dephosphorylation cascade that ultimately transduces the signal to the key transcription factors BES1 and BZR1. The dephosphorylated BES1/BZR1 transcription factors may directly bind to the ‘BR-response element’ (BRRE) in the promoters of target genes, thereby initiating their transcription [64,65]. For example, 2,4-epibrassinolide (EBR) treatment significantly upregulates the expression of *CaSOS1*, *CaHKT1*, and *CaSOD* in pepper (*Capsicum annuum* L.) [66]. In addition, BR applications have been shown to reduce the severity of damages caused by salt stress in peppermint (*Mentha piperita* L.) plants. Specifically, BR positively influences the production of secondary metabolites in salt-treated peppermint [67]. Auxin also plays a crucial role in the plant’s response to salt stress. Under salt conditions, endocytosis of PIN2 and asymmetric distribution of AUX1 lead to root bending toward the low-salt side, while also upregulating local growth hormone synthesis genes (e.g., *YUCCA*, *CYP79B2/3*), which regulate lateral root growth [12]. Salt inhibits root growth by suppressing TIR1/AFB receptors or stabilizing AUX/IAA inhibitors, thereby reducing auxin responsiveness [68,69]. Additionally, ethylene promotes growth recovery by regulating microtubule rearrangement. During salt stress, ethylene signaling participates in microtubule repolymerization and bundling, aiding cellular growth recovery in the later stages of stress [70]. Moreover, ethylene influences potassium homeostasis by regulating the expression of the high-affinity potassium transporter *HAK5*, thereby helping to maintain a more favorable K^+^/Na^+^ ratio under salt stress [71].

Jasmonic Acid (JA) plays a critical role in plant responses to salt stress through multiple mechanisms, including the activation of signaling pathways, regulation of ion homeostasis, enhancement of antioxidant defense, and interactions with other hormones like ABA. Furthermore, exogenous JA application has been shown to be an effective strategy to enhance plant salt tolerance [72]. Gibberellic acid (GA), a key regulator of normal plant growth, is highly sensitive to salt stress, with its biosynthesis significantly inhibited under such conditions, leading to growth inhibition. Maintaining or restoring GA levels is crucial for mitigating salt stress. Within the plant hormone network, GA acts antagonistically to stress hormones like ABA and SA. By adjusting this hormonal balance, plants improve their salt tolerance, optimizing the trade-off between stress responses and growth [73].

### 3.3. Plants Maintain Na^+^/K^+^ Balance Through Precise Regulation of Ion Transport, the Core of Salt Tolerance

The proton pump plays a crucial role in salt tolerance mechanisms. Plasma membrane H^+^-ATPases create a proton gradient that drives the extrusion of Na^+^ from the cell, while plasma membrane P-ATPases maintain membrane potential and facilitate Na^+^ efflux mediated by SOS1 transporter [74,75]. SOS1/NHX7 encodes a plasma membrane Na^+^/H^+^ antiporter that plays a critical role in sodium extrusion and in controlling long-distance Na^+^ transport from the root to shoot [76,77]. *SOS3* encodes an EF-hand Ca^2+^-binding protein that functions as a calcium sensor for salt tolerance [78]. Under salt stress, elevated intracellular calcium levels activate SOS3, a calcium-binding protein, which then forms a complex with SOS2, a serine/threonine protein kinase. SOS3 directs SOS2 to the plasma membrane, where SOS2 acts as the central regulator of the SOS signaling pathway. Upon activation by SOS3, SOS2 phosphorylates SOS1, enhancing its Na^+^/H^+^ antiport function and increasing Na^+^ efflux capacity. Additionally, SOS2 localizes to the vacuolar membrane, where it regulates proteins such as vacuolar H^+^-ATPase and the Ca^2+^/H^+^ exchanger CAX1, thereby facilitating Na^+^ compartmentalization into vacuoles to reduce cytosolic toxicity [79].

Within the SOS pathway, the SOS1 and SOS2 proteins are regulated by the ESCRT component FYVE4. FYVE4 enhances the phosphorylation level of SOS1 by promoting its interaction with SOS2, thereby activating the sodium ion efflux function [80]. SOS2 directly phosphorylates and inhibits the key ESCRT protein FREE1, thereby weakening FREE1’s interactions with other ESCRT components. This reshapes endocytic transport and the vacuolar system, effectively enhancing intracellular Na^+^ buffering capacity and consequently improving plant salt tolerance [81]. Photosensitive pigments also play a role in regulating salt tolerance in plants. For example, the photosensitive pigments phyA and phyB in Arabidopsis (*Arabidopsis thaliana*) enhance the activity of SOS2 kinase, thereby promoting the expression of salt tolerance genes under salt stress [82]. Ammonium nitrogen also regulates ammonium homeostasis under salt stress by enhancing SOS2 protein kinase activity, thereby promoting its phosphorylation of AMT1;1, which enhances plant salt tolerance [83]. Phosphatidic acid (PA) is generated by phospholipases and plays a crucial role in binding and regulating SnRK2 protein kinases, such as SnRK2.4 and SnRK2.10 [84]. This regulation influences various processes, including the endocytosis of the growth hormone transporter protein PIN2, mRNA degradation via VCS proteins, and Na^+^ efflux through the activation of SOS1 [12]. The SnRK2 family is categorized into ABA-dependent and non-dependent types. The ABA-dependent SnRK2s primarily regulate ABA-related transcription and stomatal closure, while the non-dependent SnRK2s are involved in regulating root growth and mRNA stability under salt stress [85].

Research indicates that in *Arabidopsis*, the AtNHX1 protein is localized to the vacuolar membrane, where it primarily functions as a Na^+^/H^+^ antiporter, mediating the active transport of sodium ions into the vacuole. This activity leads to the sequestration and accumulation of Na^+^ within the vacuole, helping to mitigate cytosolic sodium toxicity [86]. In contrast, NHX5/6, as endosomal proteins localized to the Golgi apparatus and trans-Golgi network (TGN), may enhance salt tolerance by regulating endosomal pH and influencing the Na^+^/H^+^ transporter on the vacuolar membrane [87].

**Figure 1:**
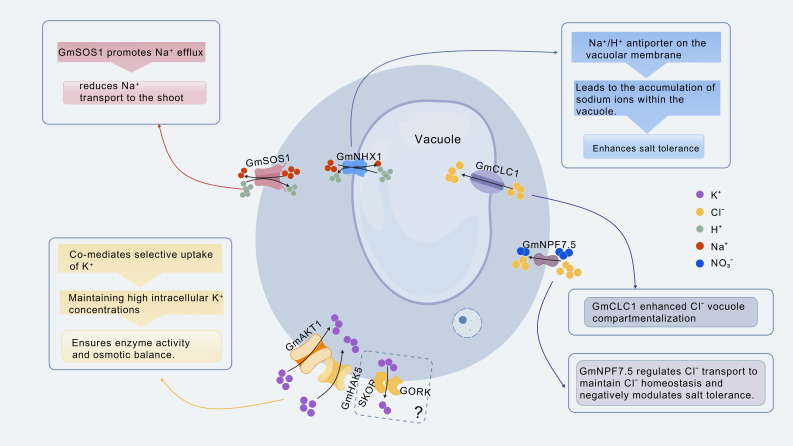
Schematic diagram of ion transporters linked to salt tolerance in soybean. Purple dots represent K^+^, yellow dots represent Cl^−^, red dots represent Na^+^, and blue dots represent NO_3_^−^. Under salt stress, GmSOS1 promotes Na^+^ efflux from roots; the GmNHX1 protein compartmentalizes Na+ from the cytoplasm into vacuoles; GmCLC1 enhances Cl^−^ compartmentalization into the vacuole; GmNPF7.5 regulates Cl-transport and negatively modulates salt tolerance; GmAKT and GmHAK jointly mediate selective K^+^ uptake, maintaining high intracellular K^+^ concentrations; GORK and SKOR jointly mediate K^+^ efflux, leading to cellular K^+^ loss; however, their roles in soybean remains unclear.

In addition, HAK5 is a high-affinity K^+^ transporter that preferentially facilitates K^+^ uptake under both low and high salt conditions. In *Arabidopsis*, the loss of *AtHAK5* impairs the plant’s ability to absorb K^+^ under low potassium conditions, particularly during salt stress [88]. Under salt stress conditions, AKT1 mediates potassium ion influx [89], whereas GORK and SKOR are responsible for potassium ion efflux (Figure 1). Notably, *Arabidopsis gork-skor* double mutants accumulate higher levels of K^+^ than wild-type plants under salt stress, providing strong evidence that GORK/SKOR contribute to K^+^ loss [90].

In plants, HKT transporters alleviate Na^+^ cytotoxicity by modulating the distribution of Na^+^ within the plant [91]. For example, in *Arabidopsis*, AtHKT1;1 unloads Na^+^ from the xylem, thereby reducing Na^+^ accumulation in the leaves [92]. In rice, *OsHKT1;5*, expressed in the xylem parenchyma cells, restricts the transport of Na^+^ to the shoot [93].

Proteins of the chloride channel family (CLC, ChLoride Channel) function as anion channels and anion/proton antiporters, playing important roles in nitrate (NO_3_^−^) and chloride homeostasis (Cl^−^) at both cellular and whole-plant levels [94]. In *Arabidopsis*, seven CLC proteins have been identified: CLCa, CLCb, CLCc, CLCd, CLCe, CLCf, and CLCg, all of which are localized to intracellular membranes. However, their specific functions are not yet fully understood [95]. Among these, CLC-b, CLC-c, and CLC-g have been suggested to localized to the vacuolar membrane, with CLC-b speculated to preferentially transport NO_3_^−^, while CLC-c and -g are selective for Cl^−^, mediating its sequestration into vacuoles [96].

### 3.4. Osmoregulation

Salt stress causes an increase in extracellular osmotic pressure. To counteract this, plants reduce their intracellular osmotic pressure by accumulating low-molecular-weight osmoprotectants within the cytoplasm, thereby maintaining water uptake and cell turgor pressure. These osmoprotective compounds include proline, sugars, and others, which serve both osmoregulatory functions and protect biomolecules from stress-induced damage [54]. Proline, a key osmoprotectant, rapidly accumulates under salt stress, helping to reduce water loss by lowering cytoplasmic osmotic potential and stabilizing protein structures [97]. Similarly, sugars such as sucrose, fructose, and inositol increase in concentration during salt stress and contribute to osmoregulation. Other compounds, including betaine and trehalose, further support osmotic regulation by stabilizing cell membranes and enzyme activity [98].

Osmotin plays a crucial role in the response to salt stress. Tobacco osmotin (Tbosm) significantly enhances soybean tolerance to salt stress by promoting proline accumulation, facilitating osmotic adjustment, activating the antioxidant system, protecting photosynthetic functions, and improving cellular water retention capacity [99].

In addition to accumulating osmoprotectants, directly reducing the transpiration rate in plants is an effective strategy to limit water loss under salt stress. For example, overexpression of the wheat aquaporin gene *TdPIP2;1* has been shown to enhance salt tolerance by improving water use efficiency and osmotic adjustment [100].

## 4. Molecular Pathways Involved in Salt Tolerance in Soybean

### 4.1. Ion Homeostasis

Under salt stress, excessive Na^+^ and Cl^−^ disrupt cellular functions [101]. Soybean maintains intracellular ion homeostasis through the coordinated activity of ion-transporting proteins (Figure 1). The sodium-hydrogen antiporter GmNHX1, located on the vacuolar membrane, transports excess Na^+^ from the cytoplasm into the vacuole, thereby reducing cytoplasmic toxicity. Studies have shown that overexpression of *GmNHX1* enhances salt tolerance by promoting Na^+^ sequestration into vacuoles [102]. Meanwhile, the plasma membrane–localized GmSOS1, a homolog of *Arabidopsis* SOS1, found on the plasma membrane, is proposed to facilitate Na^+^ efflux from the roots, thereby reducing Na^+^ transport to aerial tissues [103].

Chloride (Cl^−^) channels located on the vacuole membrane transport Cl^−^ into vacuoles, effectively reducing cytoplasmic Cl^−^ accumulation. Overexpression of *GmCLC1* in transgenic tobacco BY-2 cells has been shown to enhance vacuolar compartmentalization of Cl^−^, thereby improving cell viability under salt stress [102]. Meanwhile, a recent study identified a NITRATE TRANSPORTER 1 (NRT1)/PEPTIDE TRANSPORTER family (NPF) protein, GmNPF7.5, as the dominant gene locus influencing Cl^−^ homeostasis and negatively regulating salt tolerance in soybean. GmNPF7.5 can transport Cl^−^ and NO_3_^−^, but it shows a preference for Cl^–^. Furthermore, GmPI4Kγ4 interacts with and phosphorylates GmNPF7.5, enhancing soybean salt tolerance by inhibiting Cl^–^ transport without affecting NO_3_^-^ permeability [104].

Additionally, the inwardly rectifying K^+^ channel (GmAKT1) facilitates K^+^ uptake, ensuring high intracellular K^+^ levels, which are essential for enzyme activity and osmotic balance [89]. In soybean, the plasma membrane-localized potassium transporter GmHAK5 also plays a key role in K^+^ uptake by transporting K^+^ into the cell, contributing to the maintenance of elevated intracellular K^+^ concentrations [105]. Notably, salt-tolerant varieties sustain higher K^+^/Na^+^ ratios under salt stress, which supports their enhanced tolerance.

Concurrently, GmHKT1;1 functions as a Na^+^-selective transporter with low affinity for K^+^, demonstrating typical sodium specificity. This property allows GmHKT1;1 to primarily mediate Na^+^ transport rather than Na^+^/K^+^ cotransport. Under salt stress conditions, such selectivity is critical for excluding or compartmentalizing Na^+^ from sodium-sensitive tissues, such as leaves [106].

### 4.2. Osmotic Adjustment

Salt stress leads to cellular water loss, prompting soybean to maintain osmotic balance by accumulating osmoprotectants and synthesizing stress-related proteins. Although soybean has a limited capacity for glycine betaine synthesis, exogenous application has been shown to enhance photosynthetic efficiency, nitrogen fixation, and yield, while also stabilizing proteins and membrane structures [107,108]. Proline accumulation, observed in some salt-tolerant species, may aid water uptake by lowering osmotic potential; however, findings across studies have been inconsistent, potentially due to differences in genetic background and stress severity [109]. The salt-tolerant soybean variety ‘Forrest’ maintains osmotic balance by accumulating trigonelline (TRG), a response not seen in the sensitive variety ‘Essex’ [110]. Furthermore, under drought and salt stress, pinitol content in soybean leaves increased significantly. Soybean germplasm from arid or semi-arid regions also exhibits higher basal pinitol levels, suggesting that osmotic stress adaptation involves regulation of genes related to pinitol synthesis [111].

Late embryogenesis-enriched proteins (LEA proteins) are a class of hydrophilic, heat-stabilized proteins [5]. Induced expression under salt stress, it exerts its effects through three mechanisms: as an antioxidant to scavenge ROS, to stabilize membrane and protein structures, and to maintain osmotic balance and prevent cell lysis [112]. All four types of LEA proteins were found in soybean. LEA proteins participate in the osmotic salt tolerance process of soybeans through multiple mechanisms. Group 1 and Group 2 primarily exert protective effects by stabilizing membrane structure and proteins [113]; Group 3 specifically responds to ionic stress, potentially maintaining cellular homeostasis through ion chelation [114]; and Group 4 reduces dehydration damage through conformational adjustments [115]. These proteins collectively enhance soybeans’ osmotic regulatory capacity under salt stress, constituting a crucial component of salt tolerance mechanisms. A recent study revealed that GmPM30 indirectly reinforces the function of the LEA family through protein–protein interactions within the GmLEA1-GmPM30-GmLEC1 module, collaboratively maintaining membrane integrity. Furthermore, GmPM30 significantly upregulates the expression of genes involved in ROS scavenging and those related to ion homeostasis and transport (e.g., *GmNHX2*, *GmCHX19*, *GmRbohB-2*), collectively enhancing soybean tolerance to salt stress [116].

### 4.3. Restoration of Oxidative Balance

Under salt stress, soybean plants accumulate excessive levels of reactive oxygen species (ROS). RESPIRATORY BURST OXIDASE HOMOLOG B (*GmRbohB*) is a key gene governing ROS production and signaling. The protein encoded by *GmRbohB* belongs to the plant-specific NADPH oxidase family and functions as the central catalytic unit for intracellular H_2_O_2_ synthesis, providing a molecular basis for stress signal transduction [117]. The accumulation of ROS, in turn, activates the plant’s antioxidant system to scavenge these reactive oxygen species and mitigate oxidative damage. In salt-tolerant varieties, activities of key antioxidant enzymes—glutathione reductase (GR), superoxide dismutase (SOD), ascorbate peroxidase (APX), and catalase (CAT)—are significantly increased. For example, the salt-tolerant variety ‘BB52’ exhibits enhanced SOD and APX activities under salt stress, accompanied by reduced superoxide (O_2_^−^) production [118]. GmAPX and GmCAT are core enzymes responsible for ROS scavenging and maintenance of cellular redox homeostasis. These two enzymes cooperate synergistically to cope with biotic/abiotic stresses, as well as regulate plant growth and development. GmAPX relies on ascorbic acid for efficient ROS scavenging, whereas GmCAT is a heme-containing redox enzyme that can directly catalyze the decomposition of H_2_O_2_ without requiring additional electron donors [119]. Soybean mitigates lipid peroxidation induced by salt stress by regulating the expression of specific enzymes. For instance, the mitochondrion-localized purple acid phosphatase (GmPAP3), via its Fe^3+^ center, participates in redox reactions, effectively reducing the level of lipid peroxidation under salinity. Transgenic *Arabidopsis* plants overexpressing *GmPAP3* gene exhibit enhanced salt tolerance compared to wild-type plants [120,121]. Furthermore, research has demonstrated that the expression of *GmPAP3* could enhance salt tolerance in rice. Transgenic rice plants show improved germination rates, longer shoots and roots, and higher survival rates under salt stress. These plants also exhibit increased activity of SOD and CAT, higher proline, water, and chlorophyll content compared to the untransformed control [122]. Non-enzymatic antioxidants such as ascorbic acid, glutathione (GSH), and carotenoids (Car) accumulate in salt-tolerant varieties and work synergistically with enzymatic systems to scavenge ROS [5].

Recent research has revealed that the GmAPC/C-GmPEX11C axis regulates abiotic stress tolerance in soybean. GmAPC/C, an E3 ubiquitin ligase, modulates peroxisomal homeostasis and ROS balance by ubiquitinating and degrading GmPEX11C. *Gmilpa1*/*apc8* mutant impedes the degradation of GmPEX11C and exhibited enhanced salt and drought tolerance, conversely, the enhanced salt-tolerant phenotype conferred by the *Gmilpa1*/*apc8* mutation is abolished by knocking down *GmPEX11C*. [123]. In addition, the *GmTP55* gene encodes a protein belonging to the aldehyde dehydrogenase 7 (ALDH7) family that helps maintain cellular redox balance by removing reactive aldehydes produced by lipid peroxidation under salt stress, thereby indirectly participating in osmotic regulation. This gene is induced under salt and dehydration stress, and its heterologous expression enhances tobacco tolerance to oxidative and salt stress [124].

### 4.4. Structural Adaptation

Salt gland-like structures have been identified in the leaves and stems of wild soybean (*Glycine soja*), particularly in populations near the Yellow River estuary, where they may facilitate salt excretion through secretion. These structures are uniquely configured with a “spherical head + stalk-like base”. The head cells are expanded spheres with a diameter of approximately 21.6 μm, while the basal cells are small and stalk-shaped, together forming a complete salt gland unit. Salt glands play a critical role in salt excretion in soybean plants. Mature salt glands decompose salts to release corresponding ions, thereby effectively alleviating saline–alkali stress [125]. The glandular trichomes secrete sap with high concentrations of Na^+^ and Cl^–^. When treated with salt gland inhibitors, the accumulation of Na^+^ and Cl^–^ within leaf cells increases, further supporting the role of these structures in salt regulation [126].

Additionally, salt stress triggers the expression of proline-rich proteins, which enhance mechanical strength by altering cell wall structure to better withstand stress [5]. Salt-tolerant varieties also enhance membrane stability and reduce electrolyte leakage by maintaining the phospholipid/galactolipid ratio (PG ratio). For example, the salt-tolerant variety ‘Wenfeng 7’ exhibited an increased PG ratio under salt stress, whereas the sensitive variety ‘Lianhe’ showed a decreasing trend [127]. Salt stress induces modifications in plant cell walls and modulates cell membrane lipid composition to maintain cellular structural stability [12].

### 4.5. Transcriptional Regulatory Networks

**Figure 2:**
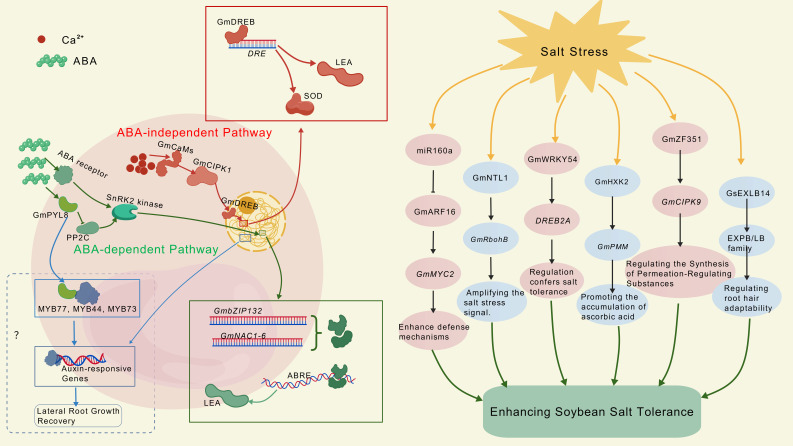
ABA signaling pathways and transcription factors that play crucial roles in mediating salt tolerance in soybean. The red dots in the left diagram represent Ca2^+^, while the green spheres denote ABA. The red portion depicts the ABA-independent pathway, where Ca2^+^ serves as the primary signaling molecule. GmCaMs receive Ca2^+^ signals and transmit them to GmCIPK1, which activates GmDREB through a cascade reaction. The activated GmDREB protein translocates to the nucleus, specifically binding to DRE cis-acting elements within the promoter regions of downstream target genes. This subsequently activates transcription of osmotic regulation genes (e.g., SOD and LEA proteins) and antioxidant genes. The green section depicts the ABA-dependent pathway: In this pathway, ABA is perceived by plasma membrane receptors, initiating intracellular signal transduction that leads to the activation of SnRK2 kinases. Alternatively, ABA binding to PYL receptors inhibits PP2C activity, which subsequently also results in SnRK2 kinase activation. The signal is then transduced to the nucleus, where the transcription factor GmbZIP132 and members of the NAC family (GmNAC1-6) bind to ABRE elements, thereby regulating the expression of LEA proteins. The blue route below the green section illustrates how PYL8 directly interacts with transcription factors such as MYB77, MYB44, and MYB73 to potentiate the auxin signaling pathway, consequently promoting the recovery of lateral root growth; however, this aspect remains subject to debate in soybean research. The right panel depicts the regulatory pathways of multiple transcription factors under salt stress.

Soybeans regulate the expression of salt-tolerance genes through complex signaling pathways, primarily involving Ca^2+^-mediated signaling, ABA-dependent and -independent pathways, as well as various cross-regulatory factors. In the ABA-independent pathway (Figure 2), initial salt stress triggers a transient increase in intracellular Ca^2+^ concentration. Ca^2+^, acting as a second messenger, is recognized by its sensor, calmodulin GmCaMs. GmCaMs transmit the signal to the downstream component cyclophosphamide kinase (GmCIPK1), thereby initiating a phosphorylation cascade. Key DREB family transcription factors GmDREB1 and GmDREB2 are activated through post-translational modifications such as phosphorylation. Activated GmDREB proteins translocate to the nucleus, where they specifically bind to DRE cis-acting elements within the promoter regions of downstream target genes. This binding directly activates transcription of osmotic regulation and antioxidant genes, including antioxidant enzymes such as superoxide dismutase (SOD) and LEA proteins [5]. Notably, transgenic tobacco overexpressing *GmDREB2* exhibits increased proline content and enhanced salt tolerance [128]. In soybeans, the overexpression of *GmDREB2* upregulates the transcription of the *P5CS* gene, which in turn promotes proline accumulation and enhances drought tolerance [129].

In contrast, the ABA-dependent pathway (Figure 2) is initiated by salt stress-induced synthesis of the stress hormone ABA. Upon recognition by plasma membrane receptors, ABA initiates intracellular signaling that is transmitted to the nucleus via SnRK2 kinases. Within the nucleus, the bZIP transcription factor GmbZIP132 and NAC family members (GmNAC1-6) regulate gene expression—including that of LEA proteins—by binding to ABRE elements [5]. These genes are crucial for maintaining osmotic and oxidative homeostasis.

In addition to the transcription factors previously mentioned, multiple genes and regulatory modules exert synergistic effects in salt tolerance in soybean (as shown in Table 1 and Figure 2). Soybeans acquire salt tolerance through a complex regulatory network composed of transcription factors and functional genes. For example, key negative regulators such as *GmARF16* are suppressed by miR160a under salt stress, thereby activating positive regulators like *GmMYC2* to enhance defense mechanisms [130]. Furthermore, salt stress induces the expression of transcription factors such as *GmNTL1* and *GmNAC06*, promoting the expression of downstream genes involved in ion homeostasis regulation and antioxidant responses [117,131]. Additionally, GmWRKY54 may confer salt and drought tolerance through the regulation of *DREB2A* and *STZ*/Zat10 [132]. Under salt stress conditions, the overexpressed GmDREB6 protein recognizes and binds to the GT-1 cis-element in the promoter of the *GmP5CS* gene via its AP2 domain. This binding significantly upregulates *GmP5CS* transcription, leading to proline accumulation and thereby achieving osmotic adjustment [133].

Salt stress also induces the expression of *GmSIN1*. The GmSIN1 protein subsequently activates *GmNCED3s* and *GmRbohBs*, thereby promoting ABA biosynthesis and ROS production. The accumulated ABA and ROS, in turn, further induce *GmSIN1* expression. This creates a positive feedback loop, ‘GmSIN1-ABA/ROS-GmSIN1’, which rapidly amplifies the salt stress signal and maintains optimal levels of ABA and ROS in the roots. This regulatory circuit ultimately promotes root growth and enhances salt tolerance [134].

Moreover, under salt stress, GmST2 directly binds to the NAC core motif in the promoters of *GmAOC3* and *GmAOC4*, thereby activating these JA biosynthetic genes and promoting JA accumulation. Key JA biosynthetic enzyme genes (e.g., *GmAOC3*, *GmAOC4*, *GmLOX*, *GmAOS*) are significantly upregulated in *GmST2*-overexpressing lines, accompanied by increased JA levels. Furthermore, GmPRL1b enhances GmST2 function by promoting the accumulation of the GmST2 protein. Together, this forms a coherent GmPRL1b-GmST2-GmAOC3/4 regulatory module that promotes JA accumulation and ultimately salt tolerance [135].

Research further indicates that GsPRX9 activates the transcription of *GsCAD* genes (e.g., GsCAD9-14g, *GsCAD9-17g*, *GsCAD4*), accelerating lignin biosynthesis. This process strengthens root cell wall compactness and stability, thereby reducing Na^+^ influx and water loss. Additionally, GsPRX9 enhances antioxidant enzyme activity to scavenge excess ROS, protecting the catalytic function of CAD enzymes and further activating stress defense responses [136].

Additionally, GmHXK2 improves antioxidant capacity by upregulating *GmPMM*, promoting ascorbate accumulation [137]. Moreover, GmZF351 directly binds to the promoter of *GmCIPK9*, activating its expression and inducing stomatal closure under drought stress to reduce water loss. GmZF351 further contributes to dual tolerance against salt and drought stress by upregulating genes related to lipid accumulation and modulating the transport of osmotic solutes [138]. In addition, GsEXLB14 confers salt tolerance by upregulating expansin (EXPB/LB family) genes and modulating root hair adaptability [139].

**Table 1:** Transcription factors involved in the regulation of salt tolerance in soybean.

Transcription Factor	Function	Downstream Genes	References
GmARF16	Salt stress induces *miR160a* to suppress *GmARF16*, thereby activating downstream salt tolerance defense mechanisms.	*GmMYC2*	[130]
GmNTL1	Establishes a H_2_O_2_ positive feedback loop, amplifying the salt stress signal.	*GmRbohB*	[117]
GmNAC06	Directly combines and activates multiple downstream functional genes, synergistically regulating ion homeostasis and reactive oxygen species balance.	*GmUBC2, GmHKT1*	[131]
GmZF351	Promote stomatal closure and prevent water loss.	*GmCIPK9*	[138]
GmHXK2	Promote ascorbic acid accumulation to enhance antioxidant capacity.	*GmPMM*	[137]
GmDREB6	Promote proline accumulation.	*GmP5CS*	[133]
GsEXLB14	Enhance the salt tolerance of soybean root hairs.	EXPB/LB family	[139]
GsPRX9	Accelerate lignin biosynthesis and enhance root cell wall compactness and stability, leading to reduced Na^+^ influx and water loss.	*GsCAD*	[136]
GmSIN1	Promoting ABA biosynthesis and ROS production.	*GmNCED3s, GmRbohBs*	[134]
GmST2	Promoting JA accumulation	*GmAOC3, GmAOC4*	[135]
GmWRKY54	May confer salt and drought tolerance through the regulation of *DREB2A* and *STZ*/*Zat10*.	*DREB2A*	[132]

### 4.6. Microbial-Mediated Mechanisms of Salt Tolerance in the Soybean Rhizosphere

Plant growth-promoting rhizobacteria (PGPR) are known to enhance growth [140,141] and induce tolerance to abiotic stresses, including salinity [142]. One study demonstrated that PGPR application enhances soybean growth, microbial diversity, and salt tolerance under salinity stress [143]. Specifically, PGPR significantly reduced Na^+^ concentration in leaves while increasing K^+^ levels in leaves, roots, and grains. Additionally, PGPR boosted carbon assimilation and soybean yield and enhanced antioxidant enzyme activities. Furthermore, PGPR enriched beneficial bacterial phyla, likely contributing to improved nutrient cycling and plant–microbe interactions, thereby further enhancing soybean resilience to salinity. Another study identified that four PGPR strains isolated from *Amphicarpaea bracteata*, a North American relative of soybean, substantially improved shoot and root growth in soybean. Among these, *Rhizobium* sp. SL42 and *Hydrogenophaga* sp. SL48 demonstrated the greatest beneficial effects on soybean growth and salinity tolerance [143].

## 5. Concluding Remarks and Perspectives

**Figure 3:**
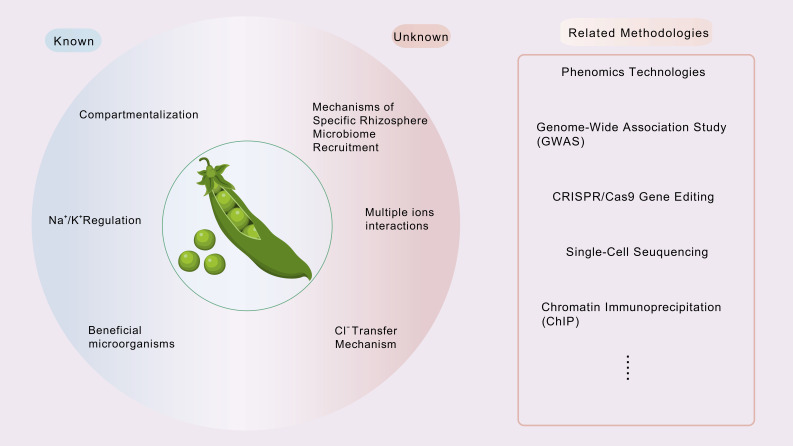
Soybean Salt Tolerance: Known and Unknown.

Compared to other major crops such as wheat and rice, soybeans are generally more sensitive to salinity, with yields severely reduced or even completely lost under saline stress conditions. Although some progress has been made in understanding the mechanisms of soybean salt tolerance, significant gaps remain compared to other crops, highlighting the need for more in-depth research (Figure 3).

In the field of ion transport, current research primarily focuses on the regulation of intracellular ion homeostasis and salt tolerance by plant sodium–potassium pumps. However, to date, little is known about the key genes and mechanisms governing Cl^–^ transport in soybeans. While multiple protein families responsible for Cl^–^ absorption and transport have been identified, such as CLC and SLAC/SLAH, further investigation is needed to clarify the specific roles of these proteins in soybeans, their differential expression among soybean varieties, and how to precisely manipulate them to enhance Cl^–^ tolerance. Additionally, although ions like Na^+^ and K^+^ have been extensively studied, the synergistic functions and transport mechanisms of other essential ions, such as Ca^2+^ and Mg^2+^, in soybean salt tolerance are relatively underexplored. Understanding how these ions interact with Na^+^ and K^+^ under salt stress to maintain intracellular ion balance could provide a more comprehensive view of the ion regulatory network involved in soybean salt tolerance. Future research should also emphasize the specificity of salt signaling, along with the spatial and temporal patterns of tissue responses and adaptive mechanisms that vary among different crops, to better enhance crop salt tolerance.

Rhizosphere microorganisms play a crucial role in regulating plant stress tolerance, yet research on the relationship between soybean rhizosphere microbial communities and salt tolerance remains limited. On the one hand, further investigation is needed to understand how soybeans recruit specific rhizosphere microorganisms under salt stress and the mechanisms through which these microorganisms assist soybeans in adapting to high-salt environments—such as by improving soil structure, modulating plant hormone levels, or enhancing plant antioxidant capacity. On the other hand, exploring strategies to utilize beneficial microorganisms through exogenous application or by manipulating the rhizosphere microenvironment—via specific biofertilizers or soil pH adjustments—could offer practical approaches for leveraging microbial technology to enhance soybean salt tolerance and improve the productivity of saline–alkali soils in agricultural production.

Significant variation in salt tolerance exists among different soybean varieties; however, the genetic basis underlying this natural variation remains largely unclear. Future research could leverage multi-omics and large-scale genome-wide association studies (GWAS), combined with comprehensive salt tolerance phenotyping of soybean germplasm collections from diverse geographical regions and ecological environments, to identify additional genetic loci and haplotypes closely linked to salt tolerance. For example, Xu et al. identified 10 SNP loci tightly linked to soybean salt tolerance through GWAS and genome-wide prediction [144]. Pruthi et al. identified key candidate genes involved in stress signaling—including *GmHAK5*, *GmGSTU19*, *GmKUP6*, *GmTDT*, *GmCHX20a*, *GmOST1*/*SnRK2.6*, *GmERF98*, and *GmERF1*—through comparative transcriptomic analysis between genotypes [145]. Despite these advances, many salt tolerance genes remain to be explored within the soybean genome. Subsequent studies should focus on elucidating how these natural genetic variations affect the expression and function of salt tolerance-related genes in soybean. This work will provide valuable genetic resources and precise molecular markers to facilitate molecular breeding efforts aimed at developing new soybean varieties with enhanced salt tolerance, tailored to thrive in various saline soil conditions.

## Data Availability

Data is contained within the article.
